# A proposed method for differentiating knives from cut marks on bone: A forensic anthropological approach

**DOI:** 10.1177/00258024231198912

**Published:** 2023-09-16

**Authors:** Melodi Ghui, Constantine Eliopoulos, Matteo Borrini

**Affiliations:** School of Biological and Environmental Sciences, 4589 Liverpool John Moores University, Liverpool, UK

**Keywords:** Forensic science, forensic anthropology, sharp force trauma, knives, cut marks, flowchart, microscopic analysis

## Abstract

Knife cut marks have been extensively studied in forensic literature, and the results have been beneficial in identifying both the cut mark and implement type on bone. For this study, an assessment in differentiating knife cut marks has been attempted by using sword cut mark characteristics. In addition, a flowchart was created to assist in interpreting such marks. Knives of different serrations (non-serrated, macro- and micro-serrated) were used to inflict cut marks on pig ribs with a forward and backward stab thrust. The analysis revealed that some traits adapted from previous studies applied to knife cut marks and that non-serrated and serrated marks could be distinguished accurately using the flowchart. The distinction between different types of serrated blades was not as successful. However, results show that further research and development of the flowchart can be invaluable in similar analyses.

Experimental skeletal trauma research has successfully identified characteristics that match a cut mark to an implement.^1–3^ In homicides, blunt and sharp force trauma are the most frequent causes of injury and death.^4–5^ A survey of the United States medical examiners’ offices found sharp force trauma to be the third most common form of trauma.^6^ Additionally, in the United Kingdom, sharp force trauma has been suggested to be the leading cause of murder,^7–8^ as knife-related assaults have increased by 9% in the last year.^9^ Hence, studying sharp force trauma (SFT) in forensic contexts is valuable to understand its impact on the human skeleton further. Several recent studies have advanced our knowledge of the processes involved in sharp force trauma.^10–13^ They have also shed more light on the different variables of injury and bone biomechanics and, in some cases, enabling the identification of the causative tool of a cut mark.^1^ Per the Daubert standard, a proposed methodology should be subjected to peer review and testing before it is accepted by the scientific community and the legal system.^14^

Several authors have analysed knife cut marks on animal bones and have utilised various traits to identify the implement used. However, a standardised set of characteristics and terminology needs to be improved between studies in this area. Hence, the present study aims to standardise knife cut mark characteristics for identifying and differentiating weapon types. The criteria used in this research were adapted from studies on sword and saw cut marks due to the similarities between both forms of sharp force trauma.^15–16^

Moreover, this study presents a flowchart (Figure 1) designed to assist in the knife-type determination process for forensic analysis and training practitioners in the field, e.g., forensic anthropologists, physicians and crime scene officers.

**Figure 1.**
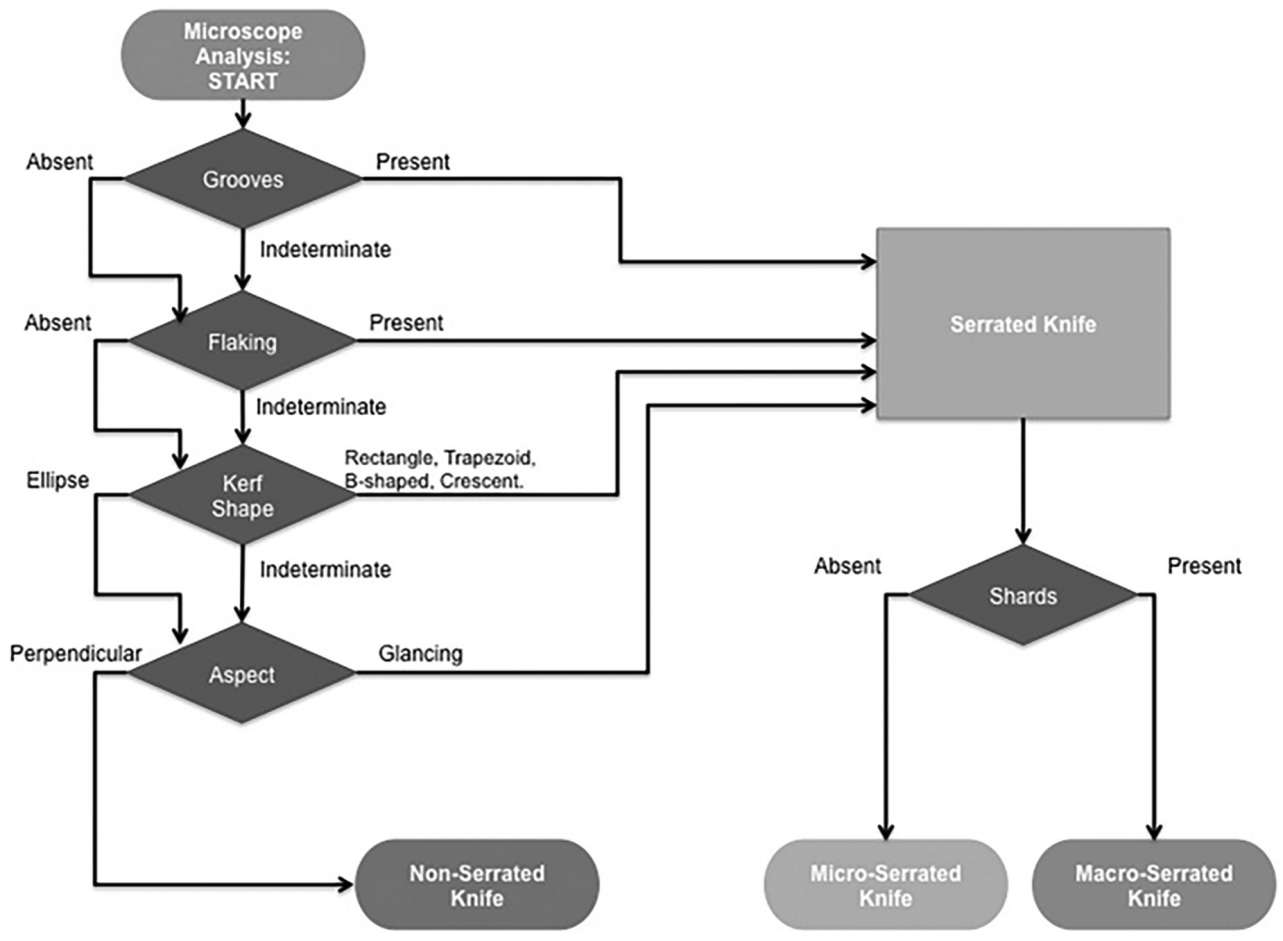
The flowchart for differentiating between knife types.

## Materials and methods

Domestic pig (*Sus scrofa*) rib bones were purchased from local butchers: no animals were killed for the present study. Porcine bones were selected for their suitability as substitutes for human rib bones, mainly due to their similarities in morphology, mineral density and macrostructure.^8,17–18^ However, choosing to utilise porcine bones could present an aspect that may affect the results. For example, their denser trabecular matrix leads to difficulty in identifying cut-mark characteristics.^17,18^ Likewise, as a proxy for human bone, pig bone is similar but not identical in exhibiting cut marks that would be present on the human skeleton. Ribs were specifically chosen, as the thorax is a common site of stabbing incidents and has been used frequently in forensic anthropological research.^8,19–21^ Before the experiment, the manually defleshed specimens were macerated in untreated tap water.^22^ The bones were then left to dry, numbered and labelled to guarantee precise data recording.

The authors categorised the 15 knives into two broad groups: non-serrated (5 knives) and serrated (10 knives). Serrated knives were differentiated further into macro-serrated and micro-serrated (5 knives each) according to the width between each serration, calculated in teeth per inch (TPI).^23^ This measurement was taken using a standard digital calliper; the average width of macro and micro-serrated knives was 5 TPI and 8 TPI, respectively.

All the knives used in the present study had one bevelled edge on the left side of the blade. A metal table clamp was used to ensure that the bone was stabilised before cuts were made swiftly forward and backward, perpendicular to the edge of the bone. The knife was rinsed under untreated tap water between each cut mark to remove any bone fragments. This was performed to ensure that no fragments would be left in the following cut, which could result in misinterpretation during analysis. Each knife was used to make ten cut marks on the ribs, resulting in 150 cut marks. The first author inflicted these cut marks, using her right hand to strike the bone along the cranial and caudal edges of the bone.

**Figure 2.**
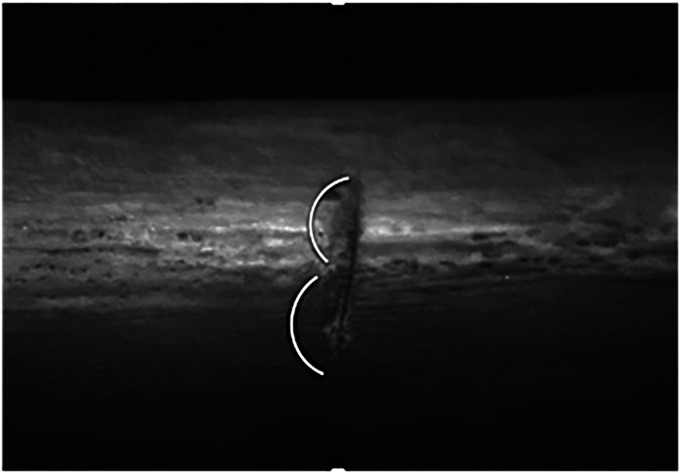
Example of grooves along the kerf wall.

All nine traits used in the present study are described in Table 1. The majority of these have been based on the definitions by Lewis.^15^ However, two new characteristics have been identified by the authors of this study: the “B-shape” of the kerf (a subcategory of trait 9) and grooves along the kerf edge (trait 3, Figure 2). Additionally, the crescent shape of the kerf has been added as another subcategory of trait 9. Although the crescent shape incision was identified by Ferllini^27^ previously, the assessment of the shape in differentiating between the knife types was not clarified in her study.

**Table 1.** The nine traits adapted and used for microscopic analysis.

Trait		Description of Trait
Trait 1	False starts	Normally used as a trait in saw cut marks, false starts are multiple cuts with an established kerf or cut in the bone.^23^
Trait 2	Shape of the kerf at cross-section	The shape of the cut mark produced when viewed cross-sectionally. The cut can include one of the following four shapes: V, U, W, Y.^15,24–26^
Trait 3	Grooves along the kerf edge	The presence or absence of grooves along one edge of the kerf wall, typically on the bevelled side of the knife.
Trait 4	Flaking	The breakage of bone that is flake-like during the forward and backward motion of the blade.^15^
Trait 5	Feathering	Small, “wispy” damage to the surface, sides or edges of the cut mark.^14^ Also known as lateral pushing back, where the edge of the wall of the kerf is “pushed back”.
Trait 6	Location of feathering	Bilaterally or unilaterally (left or right according the anatomical location of the cut).
Trait 7	Shards	Tiny splinters within the kerf of the cut mark.^15^
Trait 8	Aspect of entry	The aspect, or angle of entry can be perpendicular (symmetrical) or glancing (asymmetrical).^15^
Trait 9	Kerf shape at the cut mark	Five unique shapes that can be seen when viewed cross-sectionally: Ellipse, rectangle, trapezoid, B-shaped, crescent-shaped.^15,27^

Two other traits from Lewis’ study, breaking and cracking, are not included in the present study. These characteristics seemed inapplicable to knives because a sufficient amount of force and blade weight is needed to cause such damage to bones.

According to common procedures for tool mark analysis,^8,28–30^ all cut marks inflicted on the ribs were examined and evaluated with a stereo-zoom microscope. In this case, a Microtec (10^7^×10^45^) with a Microtec 5 mg CMOS colour camera (TEC Microscopes LTD, Somerset, England) was used.

## Results

To ascertain whether the traits identified in this study correlate to the different categories of knives, statistical tests such as Χ^2^ tests were conducted. The statistical analysis of the nine traits showed that they are all linked to the various knife types. Four traits were chosen as they best differentiated between non-serrated and serrated knives. These four traits are shown in Table 2. Trait 7, shards, was chosen to differentiate between micro and macro-serrated knives (Table 2).

**Table 2.** Traits chosen for differentiation between knife types.

	Trait	X^2^ and p-value
Non-serrated vs. Serrated knives	Grooves along kerf edge	X^2^ = 55.98, p = 5.991
	Flaking	X^2^ = 18.55, p = 5.991
	Kerf shape	X^2^ = 145.54, p = 5.991
	General aspect	X^2^ = 159.42, p = 5.991
Micro vs. Macro- serrated knives	Shards	X^2^ = 25.91, p = 5.991

As an additional tool in the classification of cut marks, a flowchart was developed based on the accuracy of each trait. The flowchart aims to enable the classification of blades used to inflict trauma on the bone from the microscopic morphological characteristics of the cut mark.

As a final test, the accuracy of the flowchart was checked by performing a blind test on 30 additional cut marks made by two external participants (one male and one female, both using their right hand). Using the flowchart, the first author assessed the cut marks without prior knowledge of which blade was used. Additionally, ten observers were selected among Liverpool John Moores University (LJMU) forensic anthropology undergraduate and postgraduate students. These participants had different levels of experience in bone analysis but no prior knowledge and training on cut marks.

They were subjected to a similar blind test of 100 cut marks using the original set of cuts. A microscopic assessment of these cut marks was made using the flowchart, and the definitions of the traits were given to assist in identifying the knife type used.

The outcome of the blind test revealed that the accuracy in differentiating between non-serrated and serrated knives ranged from 61–90%. However, the accuracy of discriminating between the two types of serrated knives was lower at 28–56%. Overall, postgraduate students performed better at linking the knife type to its resultant cut mark.

## Discussion

Several studies in the past have successfully identified knife blade characteristics on bone.^8,16,25,29–33^ Some of these have compared traits not included in this study, such as the length or width of the cut mark. These were excluded from the present study due to the lack of consistency related to the amount of force, angle, and motion of the implement, as well as the porosity of the bone. The present research has selected traits that performed well when tested and provide a high accuracy rate of distinguishing between non-serrated and serrated knives.

This study demonstrates not only that there is a correlation between the characteristic of the marks left by a blade on the bones but also that some of them are more diagnostic than others. The most indicative traits for knife-type determination are the shape of the kerf mark at the cross-section, flaking, feathering, the aspect of entry, grooves along the kerf edge and kerf shape. As a characteristic in differentiating between serrated knives, the presence of shards in the kerf can confirm whether a micro-serrated knife was used. However, a small number of macro-serrated inflicted kerfs was found to have shards. This anomaly may account for the low accuracy of the differentiation between micro and macro-serrated blades. To increase the accuracy and reliability of the classification, more traits should be identified, such as shard detail, angle or other traits. Once these have been tested and proved accurate, they should be introduced as additional steps in the flowchart. It is also important to note that the participants involved in the accuracy test had no prior knowledge of cut mark analysis, which reasonably affected the value of the results. For this reason, accuracy is expected to increase if experienced individuals perform the tests. However, considering the observers’ limitations, the results seem promising and supportive of the role of the flowchart as a valuable teaching tool.

Before starting the experiments, it was hypothesised that the results from this study would be dissimilar to those in the study by Lewis.^15^ This was assumed due to the utilisation of different implements (knife vs sword) and motion (i.e., stabbing vs chopping). However, it was found that some of the traits are transferrable from one category (sword) to the other (knife). Despite the differences in size, weight and ability to inflict a cut mark on bone, the results from the independent and blind tests have shown that the function of the implement is negligible when interpreting cut marks. Using some of the traits described by Lewis^15^ and other sources,^8,15,25,34^ we could differentiate between non-serrated and serrated knives.

## Conclusions

Despite the differences between swords and knives, the results of this study have shown that the traits from sword cut marks can be modified, adapted and identified in knife cut marks. These traits can also differentiate between non-serrated and serrated knives and supplement other features utilised in the field. Identifying a new characteristic provides an additional distinct marker in differentiating between non-serrated and serrated knives as well, even if improvement on the number and quality of traits is required in this specific field of research. Moreover, this study has explicitly shown that using a flowchart has proven effective and valuable and can be employed as a tool in performing such analyses.

Even though the results in differentiating between micro- and macro-serrated knives are promising, the authors agree that further work should be conducted to increase the degree of accuracy. It is hoped that the flowchart can be used as a teaching tool and by practitioners in examining cut marks on bone.
